# Shining a light on parasite behaviour: daily patterns of *Argulus* fish lice

**DOI:** 10.1017/S0031182021000445

**Published:** 2021-06

**Authors:** Rhi Hunt, Jo Cable, Amy Ellison

**Affiliations:** 1 School of Biosciences, Cardiff University, Cardiff, CF10 3AX, UK; 2 School of Natural Sciences, Bangor University, Deiniol Road, Bangor, Gwynedd LL57 2UW, UK

**Keywords:** Aquaculture, circadian, fish lice, parasite behaviour

## Abstract

Parasites display a wide range of behaviours that are frequently overlooked in favour of host responses. Understanding these behaviours can improve parasite control through a more precise application or development of new behaviour-based strategies. In aquaculture fish lice are an ongoing problem, infections reduce fishery production and control options are limited. Fish lice are distinct in their ability to survive and swim off hosts, allowing the transmission to multiple fish hosts across their lifespan. Here we assessed the off-host behaviour of *Argulus foliaceus* (a freshwater fish louse) and observed a diurnal rhythmical pattern in their behaviour. This pattern was lost when lice were exposed to constant darkness, indicating that the behaviour is not endogenously driven. Males were consistently active in light with reduced activity in darkness. In contrast, females were active during light and dark phases with peak activity at the start of dark periods. *A. foliaceus* was also strongly attracted to a light stimulus, preferring white- and blue-coloured lights over green- or red-coloured lights. Light is a strong driver of fish louse activity and could be used to trap parasites. Aquaculture light regimes could also be altered to reduce parasite attraction and activity.

## Graphical abstract

**Figure:**
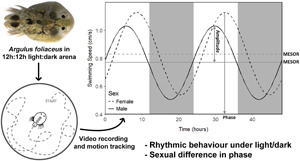

## Introduction

Parasites are a fundamental component of ecosystems; practically all known species carry parasites and food webs can be dominated by their presence (Marcogliese and Cone, 1997; Poulin and Morand, 2000; Lafferty *et al*., 2006; Dobson *et al*., 2008). In addition to their ecological importance, parasitic infections play a critical role in the global health of humans and both domesticated and wild species. The conflict between humans and parasites drives the development and use of control strategies to prevent and reduce the health and socio-economic impacts of infection. Understanding behaviour can aid the development and employment of control strategies, but research tends to focus on the host rather than parasite behaviours (Barnard, 1990; Sukhdeo and Chappell, 1994; Lewis *et al*., 2002). This is despite the fact that parasites have developed a wide range of complex behaviours to facilitate transmission, infection, reproduction and survival (Rea and Irwin, 1994; Sukhdeo and Chappell, 1994; Lewis *et al*., 2002). Behaviours involved in host finding are of particular interest regarding the development of control strategies to interrupt and prevent infection. Many parasites have adopted ‘active’ host finding behaviours to locate suitable hosts, whereby a parasite responds to environmental and/or host signals (Rea and Irwin, 1994). Parasites utilize a range of stimuli (such as chemical, thermal, mechanical and visual), often in combination to locate hosts and assess their suitability (Van Leerdam *et al*., 1985; Ashton *et al*., 1999; Bailey *et al*., 2006; Mordue (Luntz) and Birkett, 2009).

Organisms can temporally synchronize to their environment by detecting and responding to external cues, resulting in biological rhythms of physiology and behaviour (Vitaterna *et al*., 2001; Bell-Pedersen *et al*., 2005). Light–dark cycles are the dominant cue for a majority of organisms, however for parasites both environmental and host cues influence rhythmicity (Bell-Pedersen *et al*., 2005; Reece *et al*., 2017). By synchronizing with hosts, parasites can increase their survival. During dispersal and transmission, rhythms allow parasites to maximize infection success by optimizing the presence of infective stages with host availability (Sukhdeo and Chappell, 1994; Bogéa *et al*., 1996). In addition, infection success and parasite survival can be influenced by fluctuations (daily and/or seasonal) in host immune responses (Martinez-Bakker and Helm, 2015; Kiessling *et al*., 2017; Carvalho Cabral *et al*., 2019). Identification of cues used by parasites and the rhythms they exhibit could help reduce infection and transmission risks; for example, by avoiding/preventing access to locations during peak parasite presence or deploying control measures at such times to maximize capture.

The environmental and/or host cues utilized by parasites can differ between life stage or sexes. While this can reduce the efficacy of broad control applications and induce bias, it can also be used for highly targeted control. Sex-specific control schemes have been employed to successfully reduce parasite populations or their vectors. Females can be targeted to reduce the next generation by directing removing reproducers, while male targeting uses sterilization and release techniques to lower population fecundity (Alphey *et al*., 2010; Epsky *et al*., 1999). Discrete sexes can be caught using sex-specific behaviours such as pheromone or food-based attraction (Epsky *et al*., 1999). These sex-specific behaviours likely lead to sex-specific rhythms, which could also be exploited to further promote control success. Sexual differences in parasite rhythms are yet to be explored (Sikkel *et al*., 2009).

Fish lice are ectoparasitic crustaceans, which are problematic worldwide in fisheries. Control options are limited, with a reduction in chemical applications due to environmental concerns and rising drug resistance (Taylor *et al*., 2005*a*; Costello, 2009). Recent developments in the control of marine sea lice capitalize on louse behaviour: lice frequently occupy the top of the water column, consequently, fishes are held at >10 m below the sea surface to reduce infection. For freshwater lice (genus *Argulus*) however, control options remain insufficient with some farmers turning to illegal options (Taylor *et al*., 2005*b*). Thus, there is a need to explore alternative, behaviour-based control methods. *Argulus* spp. are unusual in that they retain the ability to free swim throughout their life cycle with host-switching frequency, especially among male parasites as they seek female partners (Bandilla *et al*., 2008). No studies have tested for the presence of endogenous rhythms in *Argulus* spp. (or any other aquatic ectoparasitic crustacean), although a diurnal pattern is present in the strength of their positive phototaxis response (Yoshizawa and Nogami, 2008). *Argulus* spp. also react to light/dark changes with differing activity, however, this has not been observed over a circadian period or between sexes (Mikheev *et al*., 1999).

Here we examine the host-seeking behaviour of a globally problematic fish ectoparasite over a diurnal period, testing for the presence of endogenous cues. Strength of light attraction and wavelength-specific preferences are also assessed to aid control development.

## Materials and methods

### Parasite and host maintenance

*Argulus* spp. used in this study were collected from Risca Canal (Newport, UK; grid reference: ST 24344 90686) on 6 June 2018 and 7 August 2019 by hand netting naturally infected 3-spined stickleback *Gasterosteus aculeatus.* Parasites were removed from fish in the field by lifting the host fish out of the water using a net for a 10 s period; upon re-submersion into a container of freshwater, the parasite detached and was collected using a wide-bore pipette. *Argulus* spp. were transported to the laboratory off host in sealed containers of dechlorinated water. Once in the Cardiff aquarium, parasites were morphologically identified as *Argulus foliaceus* (according to Fryer, 1982) and maintained in male: female pairs on 3-spined sticklebacks collected from Roath Brook, Cardiff (ST 18897 78541; an *Argulus* spp. naïve population). Fish were infected by placing the parasites into fish-holding tanks (9 L) containing an individual stickleback to allow natural parasite attachment. All fish and parasites were maintained under a 12 h light:12 h dark cycle, with fish fed daily with *Tubifex* bloodworm. *Argulus foliaceus* were acclimated to laboratory conditions on their hosts for 1 week prior to experimentation. Parasites were not re-used within or across experiments. For the circadian rhythm experiment, both male and non-gravid female parasites were used. For the light attraction/colour preference experiments only male *A. foliaceus* were used due to higher availability of male parasites *vs* non-gravid females (as female parasites continuously produce eggs after mating and egg-baring females exhibit egg-laying behaviour when off host).

*Argulus foliaceus* were removed from sticklebacks for use in experiments using the same collection method as described above. All *A. foliaceus* were checked visually for damage before use and measured from the rostral edge of the carapace to the anterior end of the abdominal lobes using a dissecting microscope at 10× magnification with a Lumenera Infinity 1 camera and Infinity Capture software version 6.5.4.

The experimental procedures in this study conform to the accepted principles of animal welfare in experimental science and used the minimum number of animals required to produce statistically reproducible results. All animal work was approved by the Cardiff University's Animal Ethics Committee, followed ARRIVE guidelines and was conducted under Home Office License PPL 303424.

### Circadian rhythm of parasite swimming activity off host

To understand how *A. foliaceus* behaves off host/during transmission over a circadian period, individual adult male and non-gravid female *A. foliaceus* (males: *N* = 22, average size = 3.93 mm ± 0.23 s.d. and females: *N* = 18, average size = 4.43 mm ± 0.44 s.d.) were placed into glass Petri dishes (10 cm diameter) filled with 50 mL dechlorinated water. The water level in the Petri dishes was sufficient to allow full horizontal movement while minimizing vertical motion for behavioural tracking. Additionally, the sides of each dish were covered with white fabric to reduce reflections and prevent visual disturbance (Mikheev *et al*., 1998). Parasites were then subjected to 12 h light:12 h dark (LD; average, 1000 lux) for 48 h, after which they were removed from the setup, given 1 day of recovery on stickleback hosts (to allow feeding/prevent starvation) before returning to the setup for another 48 h under total darkness (DD). The order of light condition (12:12 LD *vs* DD) could not be randomized as the total darkness regime would disrupt any entrained circadian rhythm, altering any tests post-exposure. The setup was completely reset between trials and light condition tests. Parasitic behaviour was recorded during the 48 h exposures *via* 24 h infrared CCTV cameras (Sentient Pro HDA DVR 8 Channel CCTV, Maplin). Every 4 h (zeitgeber time = ZT, ZT0 = 7 am, ZT4 = 11 am, ZT8 = 3 pm, ZT12 = 7 pm, ZT16 = 11 pm, ZT20 = 3 am; lights were on at ZT0 and off at ZT12) the total distance covered by the parasite and subsequent average swimming speed was calculated over a 2 min period using ImageJ version 1.51j8 (Schneider *et al*., 2012) to prepare video files for analysis and Kinovea version 0.8.27 (Ganni *et al*., 2018) to track parasite movement. The proportion of time spent swimming was obtained from Kinovea by calculating the time spent swimming at >1 mm s^−1^ (approximately one-fourth body length). Patterns of parasite activity were then assessed for a 24 h period and between 12:12 LD/DD trials to determine activity and entrainment of rhythm.

### Argulus light attraction in the presence of fish hosts

**Fig. 1.**
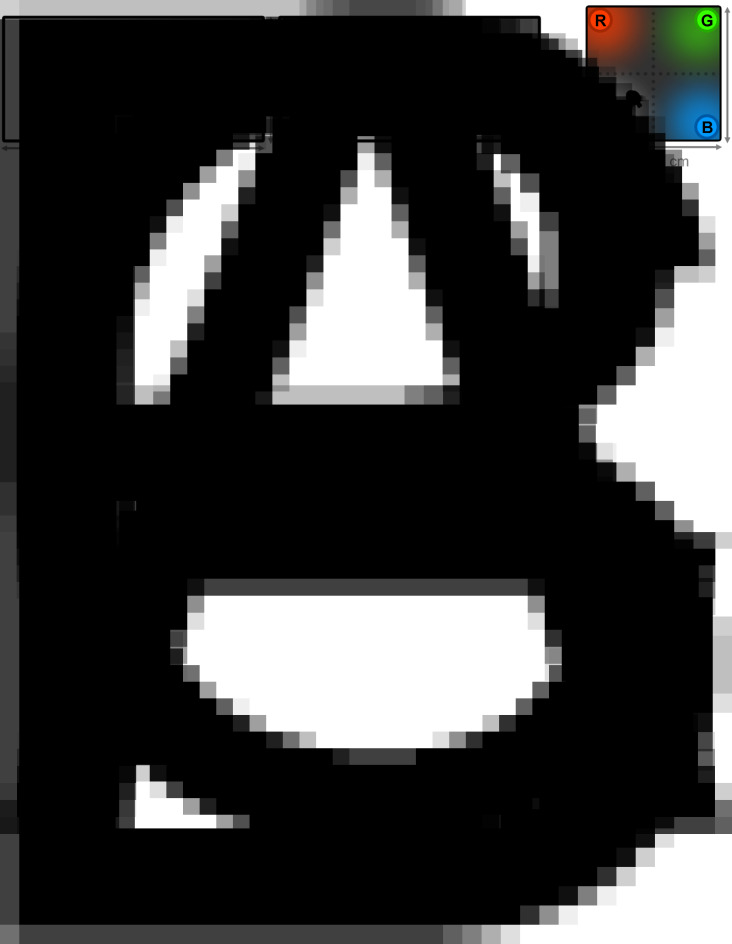
Plan view of experimental areas for *Argulus foliaceus* (A/B) light *vs* fish host preference and (C) light colour preference trials. In each arena, circles represent light-emitting diode (LED) light sources. (A) gives a choice of white light *vs* a 3-spined stickleback (*Gasterosteus aculeatus*) host with a turned-off light, (B) gives a choice of a white light + stickleback *vs* a turned off light + stickleback. In (A/B) dashed lines represent 1 cm aperture mesh which allows the parasites to swim through while blocking fish movement. In (C) dotted lines indicate the total area of each coloured corner for behavioural recording, R = red light, G = green, W = white and B = blue (coloured light placement was changed/randomized for each trial).

The attraction of *A. foliaceus* to a light source *vs* a live fish host was assessed using 2 different behavioural assays: fish *vs* light trials in which adult male *A. foliaceus* were given the choice of either white light or a stickleback in darkness over a 24 h period (*N* = 20 parasites, average size = 4.12 mm ± 0.31 s.d.; Fig. 1A), and lit fish *vs* dark fish trials offering the choice of stickleback with white light or a stickleback in darkness (*N* = 18 parasites, average size = 4.14 mm ± 0.35 s.d.; Fig. 1B) over a 2 h period. Arenas comprised of a glass tank filled to 10 cm water depth, split into 3 identical sized sections (left, middle and right) using a 1 cm aperture mesh to allow free movement of parasites while restricting fish movement (Fig. 1A and B). Stimuli were placed into the left and right thirds, with 2 *A. foliaceus* restrained under a glass dish in the middle third for 30 min to allow acclimation. After acclimation, the lice were released and monitored *via* infrared CCTV cameras. All light stimuli used a waterproof light-emitting diode (LED) white light (average 50 lux at a distance of 7 cm), while all stimuli in darkness contained the same type of LED white light but turned off to ensure each section had the same structure. The positions of the stimuli were swapped in between trials to avoid any potential side bias. For the lit fish *vs* dark fish trials, all host pairs were size matched.

### Argulus light colour preference

To investigate whether certain wavelengths of light are more attractive to *A. foliaceus,* adult males (*N* = 20, average size = 4.08 mm ± 0.33 s.d.) were placed individually into the centre of a 2.5 L opaque white square arena (14 × 14 cm) filled with 1 L water (5 cm water depth). The arena was split into 4 equal quarters, with 4 waterproof lights (3 × 3 × 2 cm, LED with RGB colour) placed into the arena and positioned to flush inside each corner (Fig. 1C). Lights were randomly assigned to emit either red (635–700 nm), green (520–560 nm), blue (450–490 nm), or white (emits all wavelengths, 450–700 nm) light, with brightness controlled so that each light individually generated an average 50 lux (lux meter positioned 7 cm away from light). There was no visual overlap in the colours emitted from each light, and initial testing found that parasites did not swim erratically or behave in any other abnormal manner in the experimental arena (following previous observations in the lab and by Mikheev *et al*., 1998). The inclusion of an acclimation period in initial testing also had no impact on parasite behaviour, thus parasites were observed immediately after introduction to the arena. After being introduced to the centre of the arena, parasites were monitored for 2 min with their time at each colour recorded. Location at a colour was classified as the parasite being present anywhere in the quarter containing the light (with more than half of the body of the parasites being present in the quarter for when the parasite crossed between sections). Parasites were observed live, with the observer stationed next to the arena looking down into the tank. Room lights were turned off so the only light source during experimentation came from the lights in the arena – this provided enough light to observe parasite movement while preventing the casting of shadows into the arena from the observer. Individual parasites were tested 3 times consecutively by calculating the average time spent in each light corner. Parasites experienced a rest period of a few seconds between replicates as the arena was reset and the light position randomized for each replicate. Parasites did not linger or remain stationary on boundary lines between quarters during observations.

### Statistical analysis

All statistical analyses were conducted using R statistical software (v3.6.2; R Core Team, 2017) with the level of significance in all tests taken as *P* *<* 0.05. Models were refined through stepwise deletion of insignificant terms and AIC comparisons, with a visual examination of model plots to check standardized residuals for normal distribution and homogeneity of variance (Crawley, 2007). The following packages were used for analyses: ‘ggplot2’ to visualize data (Wickham, 2009), ‘lme4’ to run general linear mixed models (GLMMs) (Bates *et al*., 2015), ‘emmeans’ for *post hoc* analyses (Searle *et al*., 1980), ‘RAIN’ (rhythmicity analysis incorporating nonparametric methods) and ‘MetaCycle’ to determine circadian rhythmicity (Thaben and Westermark, 2014; Wu *et al*., 2016) and ‘circacompare’ to compare rhythms (Parsons *et al*., 2020). For all rhythm analysis, the time period being examined was set to 24 h.

To detect rhythmicity, RAIN was used due to its capability in detecting and accounting for asymmetrical patterns (Thaben and Westermark, 2014) alongside MetaCycle due to its inclusion of multiple methods for rhythm evaluation (Wu *et al*., 2016). The test ‘rainresult’ was used to examine patterns across parasite sex and light condition by examining phase and peak shape. The phase of a rhythm refers to the time point at which a peak occurs, with peak shape the time (in this case: hours) between a peak and the next trough. Comparison of rhythms between different conditions was then carried out using circacompare to assess midline estimating statistic of rhythm (MESOR), amplitude and phase across rhythms. MESOR is a mean value adjusted for circadian rhythms, amplitude refers to ‘a measure of half the extent of predictable variation within a cycle’ (Cornelissen, 2014; Otsuka *et al*., 2016). A GLMM using only the 12:12 LD data was then conducted to compare activity at each ZT time point by examining *A. foliaceus* activity against ZT time, parasite sex and length with an interaction between ZT time/parasite sex. This GLMM was then repeated using the DD trials only. All GLMMs used parasite ID as a random factor to account for pseudoreplication. To determine *A. foliaceus* colour preference, a general linear model was used to compare swimming activity (average over 3 trials) against light colour and parasite length. Across all tests and trials, parasite length had no significant impact and is thus not reported further.

## Results

### Circadian rhythm of parasite swimming activity off host

**Fig. 2.**
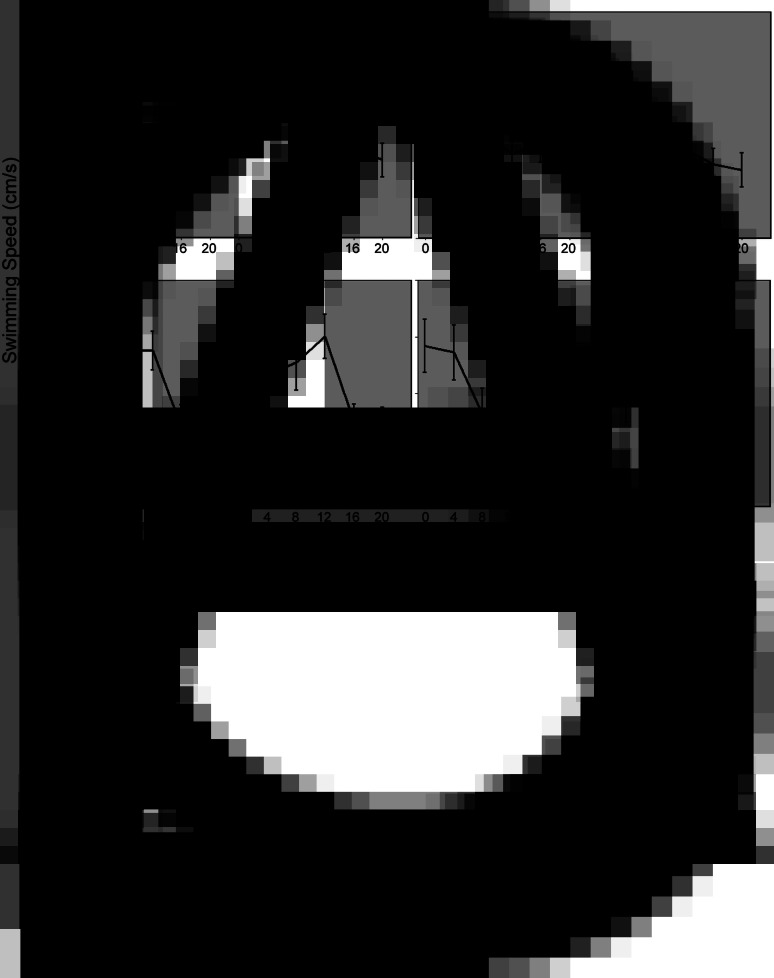
Average swimming speed of *Argulus foliaceus* off host over a 48 h period under 2 different light conditions: alternating light and dark (A and C) and total darkness (B and D). (A) Male *A. foliaceus* under 12 h light:12 h dark. (B) Male *A. foliaceus* under total darkness. (C) Female *A. foliaceus* under 12 h light:12 h dark. (D) Female *A. foliaceus* under total darkness. White backgrounds indicate periods of light, dark grey backgrounds indicate periods of darkness. Zeitgeber time (ZT)0 = 7 am, ZT12 = 7 pm.

**Fig. 3.**
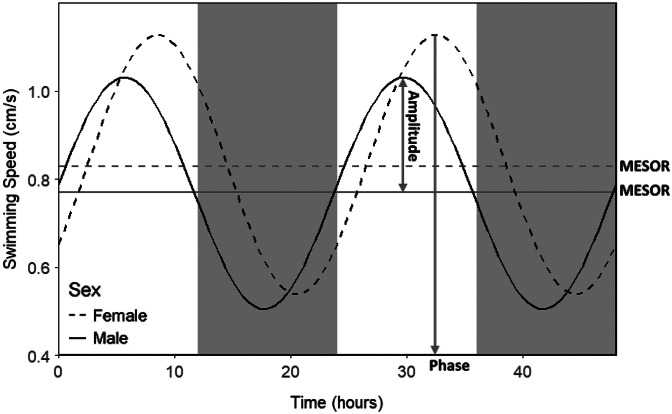
Circacompare output plot of male and female *Argulus foliaceus* swimming speed over a 12:12 light: dark 48 h period. Lights turn on/off at 0/12 and 24/36.

A strong diurnal pattern in off-host swimming activity was observed for both male and female *A. foliaceus* when maintained under 12:12 LD conditions (RAIN *P* ⩽ 0.001 for both males and females, MetaCycle *P* ⩽ 0.001/0.004 for males/females respectively; Fig. 2); however under total darkness (DD) this diurnal rhythm was lost (RAIN *P* = 0.529/0.202, MetaCycle *P* = 0.894/0.999 for males/females, respectively), suggesting this pattern is stimulated by light and not endogenously driven. Under 12:12 LD, male parasites had different phase to females (circacompare *P* = 0.018, male phase = 5.69 h post-ZT0, female = 8.56 h), but there was no difference in MESOR or amplitude (circacompare *P* = 0.290/0.716, respectively; Fig. 3).

Under 12:12 LD, the overall average swimming speed of *A. foliaceus* did not differ among sexes (0.77 and 0.83 cm s^−1^ for males and females, respectively; GLMM *P* = 0.591), however when directly comparing ZT timepoints females had a significantly higher swimming speed at ZT12 (7 pm when the lights turn off; GLMM *P* = 0.008; Fig. 2). Under DD, females had marginally significant higher overall activity than males (0.86 cm s^−1^ for females, 0.62 cm s^−1^ for males; GLMM *P* = 0.049). When examining the proportion of time spent swimming, no patterns were observed except for females under DD which showed a peak at ZT0/20 and drop at ZT8/12 (females under DD: Rain *P* = 0.005, MetaCycle *P* = 0.037, all other treatments: RAIN *P* ⩾ 0.456, MetaCycle *P* ⩾ 0.956; Supplementary Fig. 1).

#### *Argulus* light attraction in the presence of fish hosts

When assessing preference between a light stimulus or a fish host, the average time taken for lice to first enter the light section was 59 s. After 24 h, 85% of parasites were located at the light stimulus and the remaining 15% had been consumed by the fish host (time to consumption ranged from 11 to 378 s). No fish became infected during these trials.

For trials assessing preference between a fish host with or without a light source turned on, 100% of parasites moved to the section containing a fish host with a light on. After 2 h, 17% of these parasites had been eaten by the fish, 22% infected the fish and 61% remained swimming around this section.

#### *Argulus* light colour preference

**Fig. 4.**
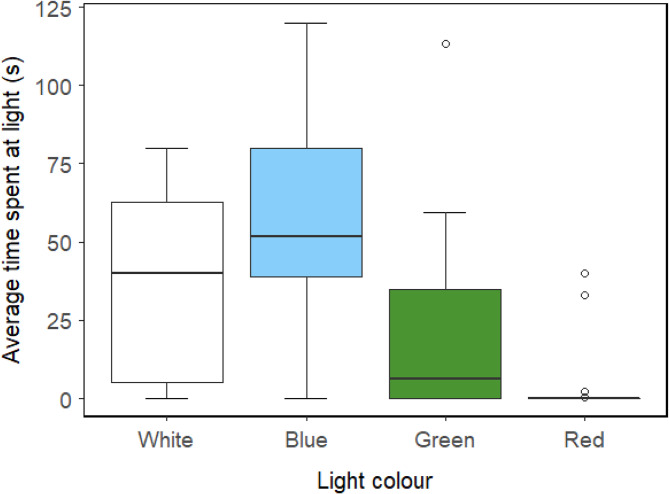
Light preference of male *Argulus foliaceus* (*n* = 20) off the host. Average time spent by free-swimming *A. foliaceus* in the vicinity of different-coloured lights over a 2-min period. Wavelengths of white light = 450–700 nm, blue light = 450–490 nm, green light = 520–560 nm, red light = 635–700 nm.

*Argulusfoliaceus* significantly preferred white- and blue-coloured lights over green- or red-coloured lights (all comparisons *P* ⩽ 0.001, except white *vs* green in which *P* = 0.025), with a preference for blue light over white close to significance (*P* = 0.052; Fig. 4).

## Discussion

During dispersal, hosts provide a spatially patchy environment in which parasites need to anticipate host availability (Skelton *et al*., 2015). As such, parasites must develop strategies to increase host–parasite contact and facilitate infection and transmission. In many parasites, this involves host-seeking behaviours and synchronization with their hosts. For fish lice, hosts are located by free-swimming parasites responding to host and environmental cues, with light being their dominant stimulus (Bandilla *et al*., 2007). While previous studies have recorded variations in fish lice behaviour over diurnal periods (Yoshizawa and Nogami, 2008; Heuch *et al*., 2011), none have determined if these rhythms are endogenously driven. Here *A. foliaceus* off-host activity followed a diurnal, not endogenous, circadian pattern as the distinct behavioural rhythm under light/dark conditions was lost under total darkness. There was also a sexual difference in off-host behaviour with male and female rhythms offset by ~4 h. When examining light attraction *A. foliaceus* consistently displayed a strong attraction to light over combined host cues (in the form of a live host) and preferred shorter wavelengths of light.

*Argulus* display sexually dimorphic host-switching behaviour with males frequently leaving their hosts to find mates while non-gravid females remain on the host (Bandilla *et al*., 2008). This dimorphism continues in off-host behaviour. As shown previously by Mikheev *et al*. (1999), female *A. foliaceus* had the highest activity when the lights turned off and low activity when lights turned on. Examining activity over a circadian period; however, indicates that this is not sustained for 4+ hours after lights turn off female parasite activity drops, and inversely 4+ hours after lights turn on female activity increases. Males do not follow the same pattern with activity consistently higher during light periods and lower during dark periods. The continued high average speed of females when lights turn off (*vs* a drop-in activity for males) could be related to their host-switching behaviours: females are not predisposed to spending time off host, and thus may not react as quickly as males to light changes. Alternatively, the lights used in this study (and Mikheev *et al*., 1999) were turned on/off immediately and could be simulating a passing shadow (a trigger of fish lice activity, Bohn, 1910; Poulin *et al*., 1990). Females could react stronger than males to potential host cues (due to a higher tendency for females to remain on the host) resulting in high activity when lights turn off. The distinct and strong diurnal rhythm observed when using average swimming speed measurements was not observed when using measurements that only record time spent active. Average swimming speed is more comprehensive accounting for variation in activity, whereas time spent active (i.e. a simple proportion of time moving or not) cannot discern these nuances and would lead to an assumption of arrhythmic behaviour. This highlights the importance of selecting the correct activity measure when assessing rhythmical patterns in behaviour.

Light is an integral component of aquaculture systems, with differing light wavelengths, intensity and photoperiods used to manipulate fish growth and maturation (Boeuf and Le Bail, 1999; Oppedal *et al*., 1999; Villamizar *et al*., 2011). The subsequent impact of these altered light regimes on both fish behaviour and health is now being considered. Recent studies have also found parasitic infection can alter host circadian gene expression, further complicating the relationship between parasites, hosts and the rhythms they both follow (Ellison *et al*., 2018, 2020). Considering the positive phototactic response of fish lice, aquaculture lights could attract lice to cages and facilitate infection (Trippel, 2010, Stewart *et al*., 2013). In this study male *A. foliaceus* were more active under light *vs* dark, suggesting lit cages would not only attract lice but also increase their activity which could lead to higher infection success. Shifting the wavelength of light used in aquaculture systems could potentially allow retention of fish manipulation while limiting the impact on pathogenic organisms. For example, when inhibiting *Salmo salar* sexual maturation to increase production, green and red light treatments used less energy *vs* white light treatments (Leclercq *et al*., 2011). Additionally, *Oncorhynchus mykiss* raised under red light showed improved growth compared to fish raised under blue or white light (Karakatsouli *et al*., 2008). Red light was the least attractive light colour to *A. foliaceus* (and *A. japonicus*: Yoshizawa and Nogami, 2008), therefore cages lit with red light could attract less parasites to those lit with shorter wavelengths. This may only be beneficial in outdoor systems where wild parasites enter containers/cages to infect fish, *vs* enclosed systems where parasites may be trapped in with the fish.

In addition to altering the light regimes in aquaculture to reduce parasite attraction and infection, light could be used to purposefully attract parasites into traps. Light traps have successfully captured sea lice in both the laboratory and field (where, in comparison, plankton tows captured none) and were suggested as a monitoring tool (Novales Flamarique *et al*., 2009). Unlike sea lice which show differing reaction strength to light across their life stages, *Argulus* spp. appear to be consistent in their light attraction from hatching to adulthood (Bai, 1981; Novales Flamarique *et al*., 2000, 2009; Bandilla *et al*., 2007). Additionally, freshwater habitats used for aquaculture are often smaller, enclosed areas (e.g. rearing ponds and raceways, recreational fishing lakes and reservoirs) compared to the ocean, potentially increasing the chance of *Argulus* spp. to encounter traps. Therefore, light traps could be more effective and feasible management tool for freshwater fisheries and aquaculture. Our findings suggest that over relatively short distances lice are strongly attracted to light, therefore future studies should examine the attraction distance of light coupled with trials in freshwater aquaculture systems to determine the efficacy of light traps in controlling lice infections.

Parasite behaviour can be complex and diverse with host cues, external stimulus and diurnal rhythms all affecting parasite activity. When developing control strategies, understanding behaviour allows a more effective application (i.e. during parasite emergence) and offers the potential for identifying new targets for control. Sexual differences are also critical to consider, as differing behaviour could lead to 1 sex avoiding control application. By understanding and manipulating parasites, the impact of infection on global health and economics can be reduced. Parasite behaviour is therefore an important component of management and should be considered for all problematic infections.

## Data Availability

Data will be made available upon request.

## Supplementary material

For supplementary material accompanying this paper visit http://doi.org/10.1017/S0031182021000445. click here to view supplementary material
