# Supplementary figures and images for: Shining a light on parasite behaviour: daily patterns of Argulus fish lice
Source: Parasitology. 2021 Mar 11;148(7):850–6. doi: 10.1017/S0031182021000445 (PMC9557303; doi:10.1017/S0031182021000445)

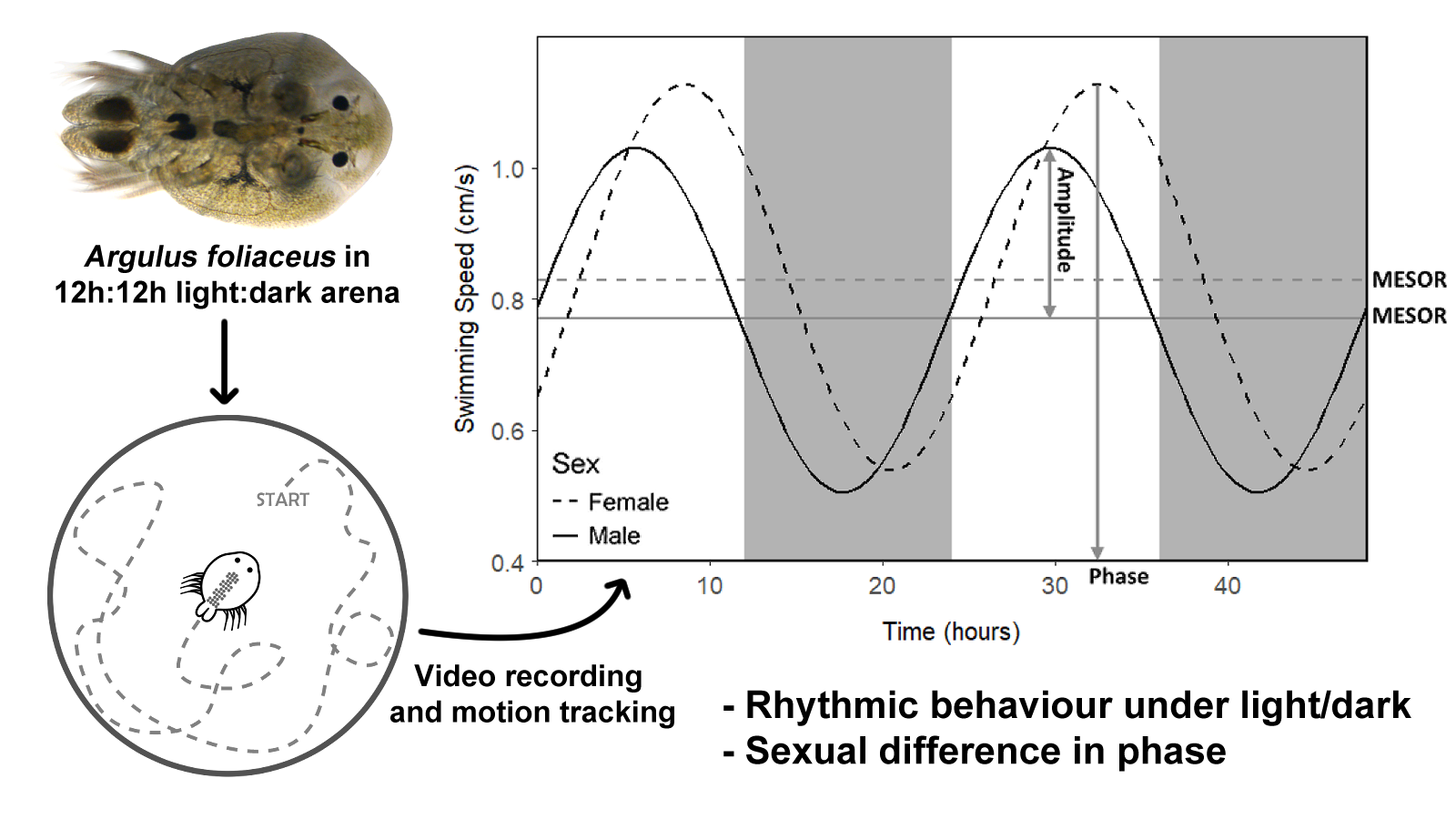

Supplement: Supplementary file 1 [file S0031182021000445sup.zip › S0031182021000445sup001.tiff]

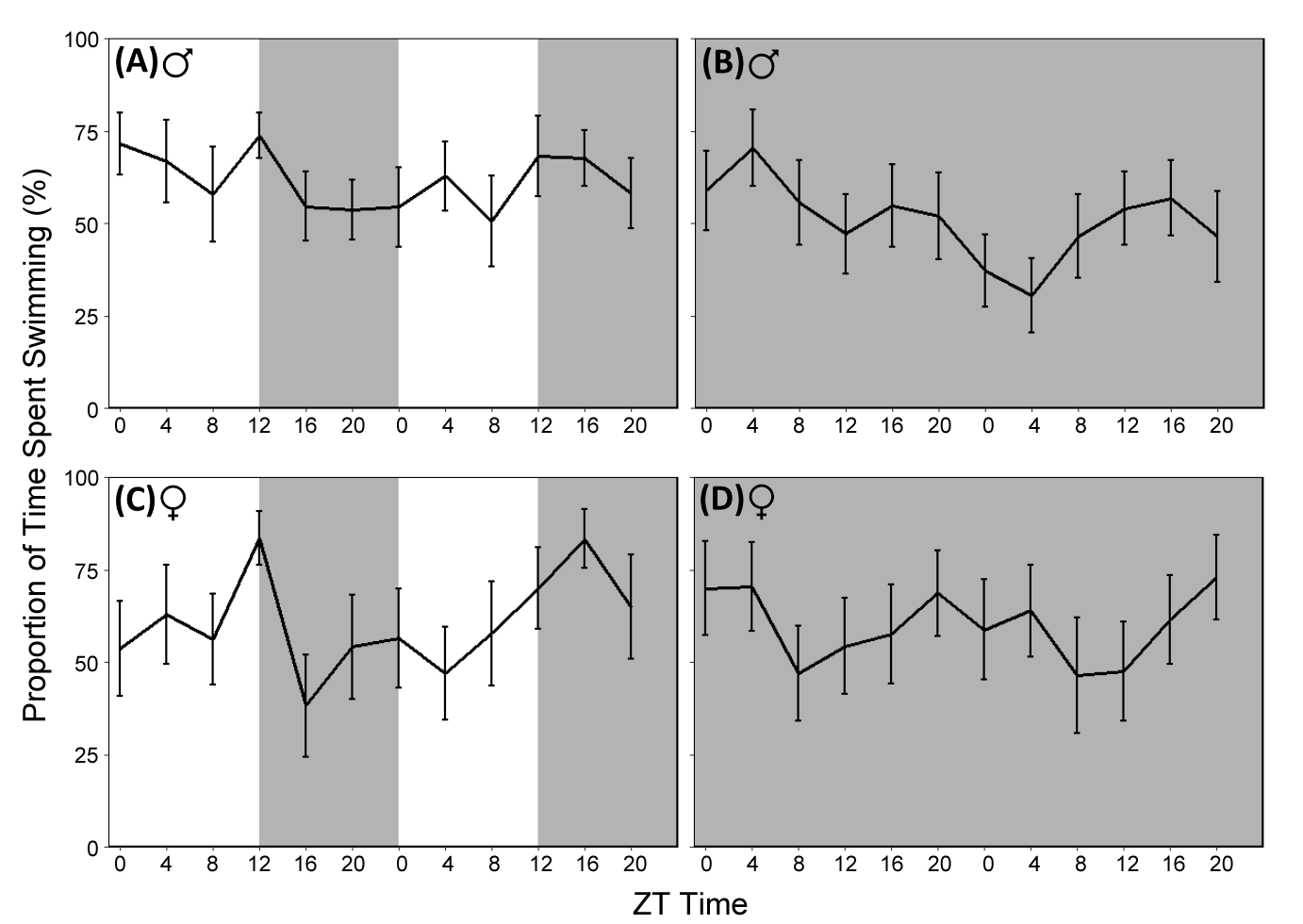

Supplement: Supplementary file 1 [file S0031182021000445sup.zip › S0031182021000445sup002.tiff]
